# Genome‐wide association study for feed efficiency in collective cage‐raised rabbits under full and restricted feeding

**DOI:** 10.1111/age.12988

**Published:** 2020-07-22

**Authors:** J. P. Sánchez, A. Legarra, M. Velasco‐Galilea, M. Piles, A. Sánchez, O. Rafel, O. González‐Rodríguez, M. Ballester

**Affiliations:** ^1^ Animal Breeding and Genetic Program Institute of Agriculture and Food Research and Technology Caldes de Montbui 08140 Spain; ^2^ GenPhySE National Institute for Agronomic Research Castanet‐Tolosan 31326 France; ^3^ Centre for Research in Agricultural Genomics Campus Universitat Autònoma de Barcelona Cerdanyola 08193 Spain

**Keywords:** candidate gene, feed efficiency, genome‐wide association study, growth, pooled records, rabbit, restricted feeding

## Abstract

Feed efficiency (FE) is one of the most economically and environmentally relevant traits in the animal production sector. The objective of this study was to gain knowledge about the genetic control of FE in rabbits. To this end, GWASs were conducted for individual growth under two feeding regimes (full feeding and restricted) and FE traits collected from cage groups, using 114 604 autosome SNPs segregating in 438 rabbits. Two different models were implemented: (1) an animal model with a linear regression on each SNP allele for growth trait; and (2) a two‐trait animal model, jointly fitting the performance trait and each SNP allele content, for FE traits. This last modeling strategy is a new tool applied to GWAS and allows information to be considered from non‐genotyped individuals whose contribution is relevant in the group average traits. A total of 189 SNPs in 17 chromosomal regions were declared to be significantly associated with any of the five analyzed traits at a chromosome‐wide level. In 12 of these regions, 20 candidate genes were proposed to explain the variation of the analyzed traits, including genes such as *FTO*, *NDUFAF6* and *CEBPA* previously associated with growth and FE traits in monogastric species. Candidate genes associated with behavioral patterns were also identified. Overall, our results can be considered as the foundation for future functional research to unravel the actual causal mutations regulating growth and FE in rabbits.

## Introduction

Before the availability of feeding devices for individual recording of feed intake (FI) of animals raised in groups, breeding programs to improve the feed efficiency (FE) of monogastric species achieved important genetic responses by using traits that could be measured individually in animals housed in groups and were genetically correlated with feed efficiency as selection criteria. In this context, FE should be understood as a general concept that reflects the degree of efficacy in the use of feed resources for performance. In the case of rabbits (Estany *et al*. 1992) and poultry (Emmerson 1997), the selection criterion is traditionally growth rate or body weight at slaughter, whereas in the case of pigs (Sather & Fredeen 1978) it is an index based on growth rate and backfat thickness. Genetic improvement of FE via indirect selection for these criteria has been possible given that they show high heritabilities and moderate correlations with direct measures of FE such as feed conversion ratio (FCR) or residual feed intake. In all of the aforementioned species, and in particular in rabbits, FE traits, jointly with prolificacy, are the most economically relevant ones (Cartuche *et al*. 2014). In addition, the improvement of FE is expected to have positive effects for decreasing the environmental footprint of the rabbit production industry (Gidenne *et al*. 2017; Cesari *et al*. 2018).

Owing to the non‐availability of electronic feeders for individual recording of FI in rabbits housed in groups, Drouilhet *et al*. (2016) performed a selection experiment to improve FE in which animals were housed individually. Despite this strategy offering an interesting framework for understanding FE from a metabolic perspective, it overlooks social interactions between cage mates, which are crucial when animals are raised in groups and especially under restricted feeding (Piles *et al*. 2017). This is a common practice in commercial rabbit farms to control digestive disorders during fattening (Gidenne *et al*. 2012). Therefore, it could certainly be argued that studies with individually housed rabbits do not reflect the reality of commercial farms where animals are reared in groups. However, this experiment provides compelling evidence of favorable genetic responses even when evaluated on animals raised in collective cages (Garreau *et al*. 2019).

The present study uses data collected from an experiment designed to estimate genetic parameters of FE in animals raised in groups. Therefore, the available information consists of weekly group records of FI and individual records of body weight (BW). Piles & Sánchez (2019) studied the data collected in this experiment from a quantitative genetic perspective, estimating heritabilities and genetic correlations of growth and FI on animals raised in groups and under either full or restricted feeding. They also proposed breeding value predictions for FE measures derived from cage‐recorded FI and individual growth and metabolic weight.

After the initial rabbit genome assembly (Lindblad‐toh *et al*. 2011), Carneiro *et al*. (2014) released an improved version with the aim of identifying domestication sweeps in rabbits. From the SNPs detected in this study, 200 000 SNPs were included in one array commercialized by Affymetrix, opening up possibilities to conduct genomic studies based on dense panels in this species.

The objective of this study was to identify genomic regions and potential candidate genes associated with traits involved in the growth and FE of meat rabbits raised in collective cages under different feeding regimes using a high‐density SNP array for this species. To achieve this objective, it was necessary first to create a model for handling collective cage performance records in the framework of GWAS studies, this being a partial objective in our study.

## Material and methods

### Animals

The animals used in this study belonged to the Caldes line (Gómez et al., 2002), and the experiment was conducted at the rabbit farm of the Institute of Agriculture and Food Research and Technology. They were randomly sampled from four batches during the first semester of 2014 and from an additional batch in spring 2016. The animals from 2014 were raised in a semi‐open‐air facility and the fattening period was from 30 to 66 days of age. Eight animals were kept in each cage. The batch in 2016 was produced on a different farm under controlled environmental conditions, which produced a better growth rate and a shorter fattening period (30–60 days of age); on this other farm six animals were kept in each cage. Beyond the farms’ environmental differences and the number of animals per cage, the recorded data and management protocols were the same in both facilities.

After weaning, kits were randomly assigned to one of two feeding regimen (FR) treatments: *ad libitum* (F) or restricted to 75% of the *ad libitum* intake (R). In order to obtain homogeneous groups regarding animal size, the kits under each FR were assigned to one of two groups based on their BW at weaning: large size (LS, i.e. kits with BW >700 g) and small size (SS, i.e. kits with BW ≤700 g). Animals from the same litter were distributed to both FRs. To obtain feed restriction to 75% of the *ad libitum* FI, the amount of feed supplied during week 1 was computed as 0.75 times the average feed intake of kits on F in a specific group *j* (*j* = LS or SS) during the previous week (i.e. *i*–1), plus 10% corresponding to the estimated increase in FI as the animals grew, i.e. ${\text{FI}}_{\text{R,}ji}=0.75\times \left(\left(1+0.1\right)\times {\text{FI}}_{\text{F,}j\left(i‐1\right)}\right)$ for *i* = 1–5 and *j* = LS or SS.

This amount of feed was multiplied by the number of animals present in the cage to determine group feed requirements. The amount of feed for week 1 was computed from data that were recorded in previous experiments on the same production line with animals raised in the same season. The actual amounts of feed provided to the restricted animals were, on average, 75 and 74% the *ad libitum* intake in LS and SS kits respectively. A maximum of two kits per litter were allocated to the same cage in order to minimize collinearity between maternal and pre‐weaning environmental effects and cage effects.

In both experimental groups (F and R), the recorded raw data consisted of weekly individual BW, and for the case of the F weekly cage, FI. In both groups, kits were fed the same standard pellet diet, supplied once per day in a feeder with three places, and containing prescribed antibiotics to control gut disorders. In both experimental facilities, feed was changed to a standard feed without drugs during the last week of fattening. Thus, records from the last week were discarded for the analysis because of the effect that the lack of antibiotics in the feed might have on growth rate, FI and the derived FE measures. Therefore, in both farms, the growing period controlled was from 30 to 56 days of age; thus, a total of four weekly individual BW records were retained per animal and three weekly group FI measurements were considered per cage.

From these raw records, individual average daily gain (ADG) was computed as the regression coefficient of the within‐animal BW records on their ages. This was done for each FR, obtaining individual ADG on *ad libitum* (ADG_F_) or restricted (ADG_R_) FR. For the 99 cages on F, individual average daily feed intake (${\bar{\text{ADFI}}}_{F}$) was computed as the total feed intake of the cage during the whole fattening period divided by the number of days and number of rabbits present in the cage during the whole fattening period. Also, individual average daily feed conversion ratio (${\bar{\text{ADFCR}}}_{F}$ **)** and individual average daily residual feed intake (${\bar{\text{ADRFI}}}_{F}$) were computed. The first was the ratio between ${\bar{\text{ADFI}}}_{F}$ and ADG_F_ cage average, and the second was the residual of a batch‐nested multiple regression of ${\bar{\text{ADFI}}}_{F}$ on the ADG_F_ cage average and the cage average of the mid fattening period day metabolic weight.

Two datasets were employed in the analyses, one containing individual growth (ADG_F_ and ADG_R_) of genotyped animals (438) and another including growth from genotyped animals as well as from all their non‐genotyped cage mates (438 + 1032). This second dataset also included cage average traits (${\bar{\text{ADFI}}}_{F}$, ${\bar{\text{ADFCR}}}_{F}$ and ${\bar{\text{ADRFI}}}_{F}$ **).** Table 1 shows the number of animals per feeding regime in cages containing genotyped animals from the five batches. In Table 2, raw statistics of the traits under study are shown. They refer to the animals mentioned before (i.e. genotyped animals and non‐genotyped cage mates).

**Table 1:** Number of individual and cage records per batch and feeding regime. Genotyped and non‐genotyped animals are distinguished for the individual records

Batch	Individuals				Cages	
	Genotyped		Non‐genotyped			
	R	F	R	F	R	F
1	28	26	68	62	12	11
2	41	35	103	84	18	15
3	58	63	190	209	31	34
4	35	59	93	124	16	23
5	46	47	50	49	16	16

F, Animals fed *ad libitum*; R, animals fed under restriction.

**Table 2:** Basic statistics for the studied traits

Trait	*N*	Mean	SD	First quartile	Third quartile	Phenotypic variance^2^, ^2^
ADG_F_ (g/day)	758	53.2	9.4	50.3	58.8	77.6
ADG_R_ (g/day)	712	35.4	8.0	32.2	40.4	55.0
${\bar{\text{ADFI}}}_{F}$(g/day)^1^, ^1^	99	151.4	17.0	141.8	162.7	289.3
${\bar{\text{FCR}}}_{F}$[(g/day)/(g/day)]^1^, ^1^	99	2.8	0.2	2.7	3.0	0.2
${\bar{\text{ADRFI}}}_{F}$(g/day) ^1^, ^1^	99	0.0	5.9	−3.3	3.4	143.8

ADG_F_, Average daily gain in rabbits fed *ad libitum*; ADG_R_, average daily gain in rabbits fed under restriction; ${\bar{\text{ADFI}}}_{F}$ average daily feed intake in rabbits fed *ad libitum*; ${\bar{\text{FCR}}}_{F}$, cage average daily feed conversion ratio in rabbits fed *ad libitum*; ${\bar{\text{ADRFI}}}_{F}$, cage average daily residual feed intake in rabbits fed *ad libitum*.

^1^ Refers to cage traits.

^2^ Estimated using model 2. For cage average records, the residual variance of the model accounts for the number of animals involved in the mean; thus, these quantities actually represent individual variation.

### DNA extraction and SNP genotyping

The DNA extraction was carried out using the NucleoSpin Tissue (250prep; Macherey‐Nagel) commercial kit from liver samples of 438 rabbits collected immediately after slaughter (66 days of age). DNA extracts were sent to an Affymetrix platform to conduct genotyping using the Axiom Rabbit Genotyping Array ‘Axiom_OrCunSNP’ (Thermo Fisher Scientific), which includes 199 692 variants. Only 161 830 were segregating in our population and, after retaining the SNPs mapped in autosomes in the OryCun2.0 assembly and applying standard quality control criteria, 114 604 SNPs were kept for further analysis. The applied quality control criteria comprised retaining animals having at least 90% of SNPs correctly genotyped, SNPs with less than 5% missing genotype data and SNPs with a MAF higher than 5%. The LD (*r* ^2^) decay pattern from our population was assessed using plink 1.9 (Chang *et al*. 2015). Prior to a pairwise LD computation, in order to reduce the computational effort, the genotype file was pruned to retrieve just one SNP every 20 kb; thus, the resolution of the obtained LD decay pattern was as low as 0.02 Mb.

### Statistical analysis

Two different modeling approaches were adopted to conduct the GWASs:

#### Regression analysis on the allele content of each SNP

This model was applied to individually recorded traits (ADG_F_ and ADG_R_) and was implemented using QXPAK (Pérez‐Enciso & Misztal 2011). The procedure implemented with this model is frequently called ‘EMMAX’ (Kang *et al*. 2010).

The general equation of this model fitting the alternative hypothesis is as follows:

(1) $$y_{\mathrm{ijklmno}}={\text{SNP}}_{\mathrm{ip}}\times \alpha_{p}+B_{j}+P_{k}+L_{l}+S_{m}+c_{n}+l_{o}+a_{i}+e_{\mathrm{ijklmno}}$$

where a particular record of a given trait under study ($y_{\mathrm{ijklmno}}$) – ADG_F_ or ADG_R_ – (one at the time) is explained by the effect of the allele content (${\text{SNP}}_{\text{ip}}$: 0, 1, 2 depending on the number of copies of the reference allele) in the *p*th genomic position of the *i*th animal, reflected by the regression coefficient at that particular position ($\alpha_{p})$ which represents the allele substitution effect, the effect of the *j*th batch level (*B_j_*, five levels), the effect of the *k*th level of the order of parity in which the animal was born (*P_k_*, four levels), the effect of the *l*th level of size of the litter in which the animal was born (*L_l_*, seven levels), the effect of the *m*th level of the type of cage [*S_m_*, two levels, cages with animals of large body weight at weaning (>700 g) or of low body weight at weaning (≤700 g)], the effect of the *n*th cage (*C_n_*), the *o*th litter (*l_o_*) and the *i*th breeding value (*a_i_*), the last three being random effects. Thus, each random factor had associated with it a variance component to be estimated. For cage and litter effects, a diagonal structure was assumed between the different levels, whereas for the additive genetic effect the numerator relationship matrix was used to define the covariance between the individuals. Finally, a diagonal normal distribution was assumed for the residual term, $e_{\mathrm{ijklmno}}$.

At each genomic position, two models were fitted by maximum likelihood, including or not (null model) the regression on the SNP allele content (1). Then, likelihood values at their maximum were compared using likelihood ratio tests. This ratio follows, under the null hypothesis, a chi‐squared distribution with 1 degree of freedom; *P*‐values were computed from this theoretical distribution.

#### Bivariate analysis considering each recorded trait, individual growth or cage records, jointly with the allele content of each SNP

This statistical model was considered as a way to perform GWASs on group mean records. With regard to individual traits (ADG_F_ and ADG_R_), the model was the same as that fitting the null hypothesis in the case of regression analysis. For explaining cage records (${\bar{\text{ADFI}}}_{F}$, ${\bar{\text{FCR}}}_{F}$ and ${\bar{\text{ADRFI}}}_{F}$), a model similar to that considered by Piles & Sánchez (2019) was adopted. The bivariate model was defined by jointly considering, as correlated traits, one performance trait at a time (either individual or cage average) and the allele content at each SNP. The equation for explaining this allele content considered only an overall mean and the additive genetic effect in addition to a residual term (Legarra & Vitezica 2015). The effect of the marker on the trait under study was estimated through the genetic covariance of both traits. Legarra & Vitezica (2015) proved that this approach is equivalent, for individual records and complete observations, to the EMMAX model that is commonly used, the main advantage being the possibility of including missing genotypes.

The model equations for the bivariate analysis fitting individually recorded traits were the following,

(2) $$y_{\mathrm{ijklmno}}=B_{j}+P_{k}+L_{l}+S_{m}+c_{n}+l_{o}+a_{1,i}+e_{1,ijklmno}$$

$${\text{SNP}}_{ip}=\mu +a_{2,ip}+e_{2,ip}$$

Note that this model was applied to a different set of individual records from that employed with model (1). In this second case, we considered individual records from both genotyped animals and their non‐genotyped cage mates.

In the case of the analysis of group records, the equations involved in the bivariate model were the following:

(3) $$\bar{y_{\mathrm{jmn}}}=B_{j}+S_{m}+{\left(\sum_{k=1}^{N_{n}}\frac{1}{N_{n}}l_{\mathrm{nk}}\right)}_{n}+{\left(\sum_{k=1}^{N_{n}}\frac{1}{N_{n}}a_{1,nk}\right)}_{n}+e_{1,jmn}$$

$${\text{SNP}}_{ip}=\mu +a_{2,ip}+e_{2,ip}$$

Group means, $\bar{y_{\mathrm{jmn}}}$, i.e. traits of interests (${\bar{\text{ADFI}}}_{F}$, ${\bar{\text{FCR}}}_{F}$ and ${\bar{\text{ADRFI}}}_{F}$), are explained by the effect of the *j*th batch level (*B_j_*, five levels), the effect of the *m*th level of the type of cage (*S_m_*, two levels) and the averages of litters (*l_nk_*) and additive genetic effects ($a_{1,nk}$) associated with the *N_n_* individuals in the *n*th cage. Litter, additive genetic and residual effects are random factors, assumed to follow normal distributions, indexed by their respective (co)variances to be estimated using an EM‐REML procedure.

Breeding values for the two traits analyzed at a time were assumed to follow a joint multivariate normal distribution of the following form:

$$a\approx N\left(0,\left[\begin{array}{cc} \sigma_{a_{1}}^{2} & \sigma_{a_{1,}a_{2}}\\ \sigma_{a_{1,}a_{2}} & \sigma_{a_{2}}^{2} \end{array}\right]⊗A\right).$$

Similarly, for the residual term, the assumed distribution was the following:

$$e\approx N\left(0,\left[\begin{array}{cc} \sigma_{e_{1}}^{2} & 0\\ 0 & \sigma_{e_{2}}^{2} \end{array}\right]⊗I\right).$$

In the case of the residual effects, a null covariance was considered between SNP allele content and the performance trait. For the case of the additive genetic effects, this covariance ($\sigma_{a_{{\text{1,a}}_{2}}}$) under the alternative model was assumed to be non‐null, representing in this case the association, at a genetic level, between breeding values for the trait of interest and the SNP genotypes. Under the null model, $\sigma_{a_{1,}a_{2}}$ was set to zero. The REML likelihood values at their maximum were used to construct likelihood ratio tests allowing exploration of the significance of $\sigma_{a_{1,}a_{2}}$ estimates. This was done by computing *P*‐values from the theoretical distribution of the ratio under the null hypothesis, a chi‐squared distribution with one degree of freedom. From this model, the estimated effect for each SNP position was calculated as a function of the estimated additive genetic covariance (${\hat{\sigma }}_{a_{1,}a_{2}}$) and the SNP frequency ($f_{p}$) (Legarra & Vitezica, 2015):

$${\hat{\alpha }}_{p}=\frac{{\hat{\sigma }}_{a_{1,}a_{2}}}{2*f_{p}*\left(1‐f_{p}\right)}$$

In the two statistical methods, multiple test correction was performed following the procedure by Storey (2002) to adjust raw *P*‐values to a positive false discovery rate of 0.05; this was done using the r package ‘qvalue’ (Storey *et al*. 2019). The adjustment was done at two different levels: first, at genome‐wide level considering all of the tests conducted; and second, within each chromosome. In the second case, thresholds for declaring significance varied across chromosomes, and they were much less strict than those applied at genomic level. To define the genomic regions associated with the analyzed traits, those significant SNPs that were less than 1 Mb apart were grouped in the same QTL region. This distance threshold was defined based on a preliminary assessment of LD as a function of the physical distance between SNPs.

### Gene annotation and functional analysis

Associated regions were annotated considering ±1 Mb around the previously defined intervals in the rabbit genome. Gene annotations were retrieved from the Ensembl Genes 98 Database with the Biomart software (Smedley *et al*. 2015) using the OryCun2.0 reference assembly. Furthermore, functional predictions of the significantly associated SNPs were performed with vep software (McLaren *et al*. 2016).

For functional categorization of the annotated genes, GOs were determined using ClueGO version 2.5.0 plug‐in of Cytoscape (Bindea *et al*. 2009). The functions assigned to the proposed candidate genes include metabolic, behavioural or immunological pathways. Orthologous human gene names were retrieved from the Ensembl Genes 98 Database for functional categorization when a rabbit gene name was not assigned to the gene stable id. Furthermore, information from the Mouse Genome Database (Eppig *et al*. 2017) and Genecards (Safran *et al*. 2002) was used to identify gene functions affecting the analyzed phenotypes.

## Results

Two modeling approaches were used to conduct a GWAS on five phenotypic traits related to individual growth and group FE using 438 rabbits genotyped with AxiomOrCunSNP (114 604 SNPs after quality control).

At genome‐wide level, after multiple testing correction, neither of the methods returned significant associations. However, when multiple test correction was applied within each chromosome, 189 SNPs (Table S1) located in nine *Oryctolagus cuniculus* chromosome (OCC) regions (3, 5, 6, 9, 12, 13, 16, 17 and 21) were declared as significantly associated with any of the five studied traits, i.e. ADG_R_, ADG_F_, ${\bar{\text{ADFI}}}_{F}$, ${\bar{\text{FCR}}}_{F}$ and ${\bar{\text{ADRFI}}}_{F}$.

**Figure 1:**
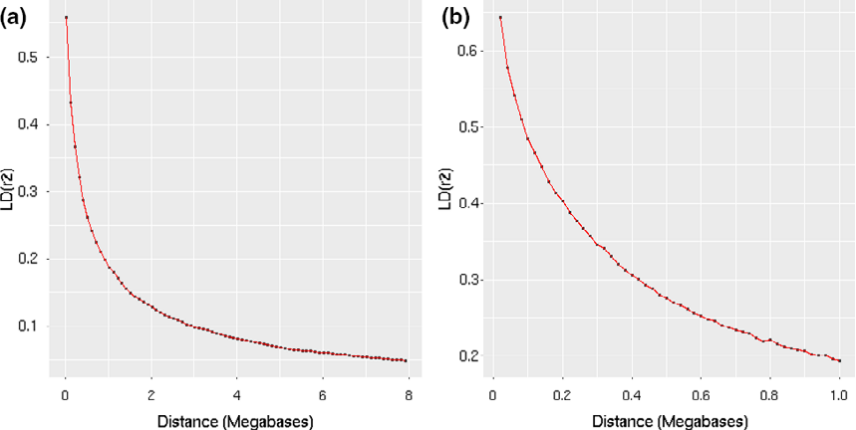
LD (*r* ^2^) decay pattern. (a) Up to 8 Mb; (b) up to 1 Mb.

It is important to describe the LD pattern decay (Fig. 1) to properly determine that the QTL intervals to be defined cover regions in relatively high LD. The LD between regions with a distance of 1 Mb was nearly 0.2. Thus, we assumed that significantly associated SNPs within a 1 Mb region pertain to the same QTL.

**Figure 2:**
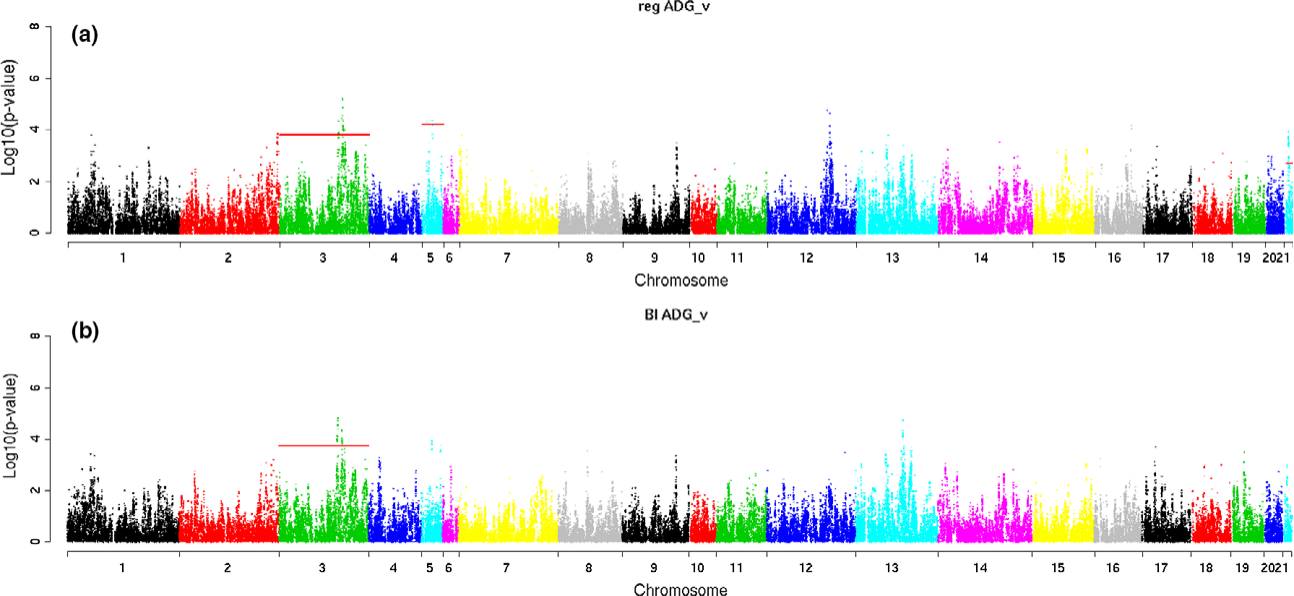
Manhattan plots for average daily gain recorded in animals fed *ad libitum* (ADG_F_) for models (a) QXPAK and (b) BI

**Figure 3:**
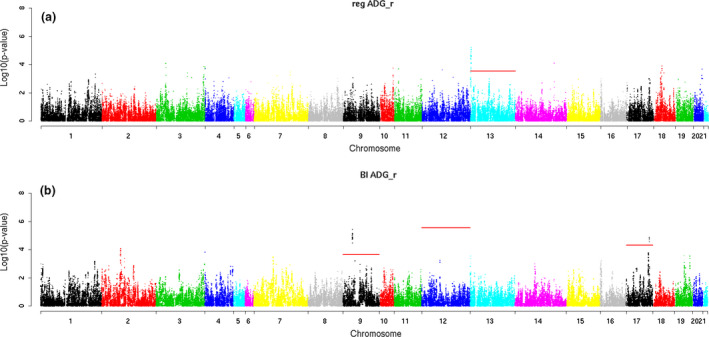
Manhattan plots for average daily gain recorded in animals fed under restriction (ADG_R_) obtained for models (a) QXPAK and (b) BI.

**Figure 4:**
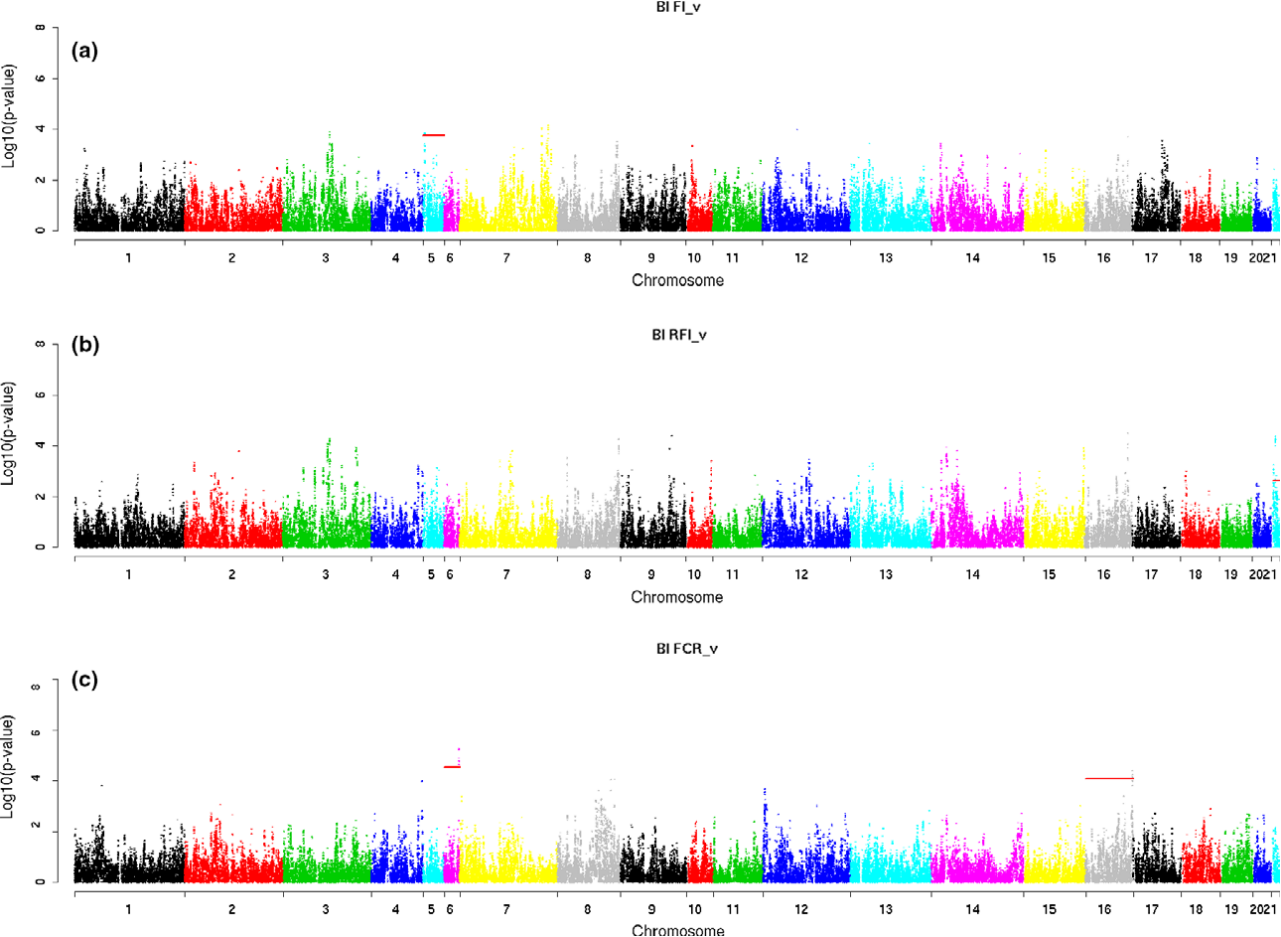
Manhattan plots using model BI for (a) average daily feed intake in rabbits fed *ad libitum* (${\bar{\text{ADFI}}}_{F}$), (b) average daily residual feed intake in rabbits fed *ad libitum* (${\bar{\text{ADRFI}}}_{F}$) and (c) average daily feed conversion ratio in rabbits fed *ad libitum* (${\bar{\text{FCR}}}_{F}$).

Table 3 summarizes the significantly associated regions with the traits of interest at chromosome level. In addition, graphical representation of the results obtained for the different traits and methods is presented in Manhattan plots (Figs 2, 3, 4).

**Table 3:** QTL regions associated with the studied traits according to the two employed methods. Effect estimates and MAF are reported

Method	Trait	Region^1^, ^1^	OCC^2^, ^2^	Initial position (Mb)	Final position (Mb)	SNPs in the region	Significant SNPs in the region^3^	SNP name^4^	*q*‐Value^5^	Effect^6^	MAF^7^
QXPAK	ADG_R_	11	13	0.40	2.09	90	50	AX‐146990063	0.0056	3.41 (g/day)	0.24
QXPAK	ADG_F_	2	3	102.22	102.37	11	9	AX‐147016699	0.0350	3.68 (g/day)	0.39
QXPAK	ADG_F_	3	3	107.99	107.99	1	1	AX‐146983203	0.0350	6.55 (g/day)	0.06
QXPAK	ADG_F_	4	3	109.07	110.88	111	16	AX‐146982129	0.0211	4.05 (g/day)	0.24
QXPAK	ADG_F_	5	3	113.46	113.46	1	1	AX‐147140896	0.0410	3.65 (g/day)	0.20
QXPAK	ADG_F_	6	5	9.07	9.07	1	1	AX‐147010974	0.0416	3.57 (g/day)	0.29
QXPAK	ADG_F_	7	5	18.95	18.97	2	2	AX‐147049894	0.0416	5.46 (g/day)	0.11
QXPAK	ADG_F_	8	21	7.17	8.46	67	26	AX‐147102744	0.0135	3.51 (g/day)	0.23
BI	ADG_R_	9	9	29.66	31.00	66	29	AX‐147167857	0.0039	1.67 (g/day)	0.07
BI	ADG_R_	10	12	99.88	99.88	0	1	AX‐146984543	0.0222	3.73 (g/day)	0.05
BI	ADG_R_	12	17	73.57	74.16	29	7	AX‐147012391	0.0183	0.95 (g/day)	0.16
BI	ADG_F_	1	3	100.99	101.11	3	4	AX‐147009110	0.0399	3.34 (g/day)	0.49
BI	ADG_F_	2	3	102.22	102.37	10	11	AX‐147016699	0.0302	3.71 (g/day)	0.39
BI	ADG_F_	4	3	109.07	109.88	58	11	AX‐147097036	0.0313	3.85 (g/day)	0.24
BI	${\bar{\text{FCR}}}_{F}$	14	6	26.28	26.44	16	10	AX‐147140966	0.0015	0.47 [(g/day)/(g/day)]	0.06
BI	${\bar{\text{FCR}}}_{F}$	15	16	82.86	83.26	26	7	AX‐147107945	0.0482	0.52 [(g/day/(g/day)]	0.42
BI	${\bar{\text{ADFI}}}_{F}$	13	5	3.70	3.85	13	12	AX‐147126724	0.0278	0.85 (g/day)	0.37
BI	${\bar{\text{ADRFI}}}_{F}$	16	21	3.89	4.33	26	8	AX‐147145784	0.0175	1.14 (g/day)	0.25
BI	${\bar{\text{ADRFI}}}_{F}$	8	21	7.16	7.70	34	15	AX‐147081855	0.0030	2.16 (g/day)	0.37
BI	${\bar{\text{ADRFI}}}_{F}$	17	21	9.21	9.21	0	1	AX‐147132637	0.0321	1.34 (g/day)	0.35

^1^ Annotated region, match to Table S2.

^2^ *Oryctolagus cuniculus* chromosome.

^3^ Chromosome‐wise *q*‐value <0.05.

^4^ Name of the most significant SNP within the region.

^5^ Within‐region minimum chromosome‐wide *q*‐value.

^6^ Absolute value of the effect of the most significant SNP.

^7^ MAF of most significant SNP.

Eight chromosomal regions located at OCCs 3, 5 and 21, were declared to be associated with ADG_F_ (Table 3 and Fig. 2). Two of the five regions on the distal segment of OCC 3 (102.22–102.37 and 109.07–109.08 Mb) were significantly associated with the trait using both modeling approaches. The estimated effects of the SNPs with the strongest association within each region ranged from 3.34 g/day (for a SNP on the region 100.90 Mb–101.11 Mb of OCC 3) when model 2 was used to 6.55 g/day (for an SNP at 107.99 Mb of OCC 3) detected with model 1. The effects of the other OCC 3‐associated regions were estimated to be close to 4 g/day. For ADG_F_, 78 ensembl_gene_ids were annotated on the declared QTL regions of OCC 3 (Table S2). One candidate gene, carbonic anhydrase 2 (*CA2*), was identified in the region 100.99–101.11 Mb, whereas two candidate genes, NADH:ubiquinone oxidoreductase complex assembly factor 6 (*NDUFAF6*) and tumor protein p53 inducible nuclear protein 1 (*TP53INP1*), were proposed for ADG_F_ in the region 109.07–110.88 Mb of OCC 3 (Table 4). In OCC 5, two significantly associated regions were detected with model 1; one region comprised a single SNP in position 9.07 Mb and the other comprised two SNPs in the region 18.95–18.97 Mb. The magnitude of the strongest estimated effects for these regions was similar to those estimated on OCC 3 (between 3.5 and 5.5 g/day). In these regions, 19 ensembl_gene_ids were annotated (Table S2). Furthermore, one promising candidate gene for ADG_F_ alpha‐ketoglutarate dependent dioxygenase (*FTO*) was identified at 9.07 Mb in OCC 5 (Table 4). Finally, one region in OCC 21 compressing 1.29 Mb (7.17–8.46 Mb) was also associated with ADG_F_. In this region, 26 SNPs were found to be significantly associated with the trait. Within this region, AX‐147049623, the SNP with the strongest association had an effect of 3.51 g/day. Remarkably, this SNP was located inside an intron of the Ataxin 2 (*ATXN2)* gene (Table S1) – one of the four candidate genes (*ATXN2*, *ACAD10*, *TRAFD1* and *PTPN11*) identified among the 71 ensembl_gene_ids annotated in this region (Table 4 and Table S2). These candidate genes contained another 10 SNPs significantly associated with ADG_F_ (Table S1).

**Table 4:** Candidate genes and their associated functions for the QTL regions declared

Method	OCC^1^	Trait	Initial Mb	Final Mb	Gene	Function	Region^2^
BI	3	ADG_F_	100.99	101.11	*CA2*	Respiration and transport of CO_2_/bicarbonate	1
QXPAK, BI	3	ADG_F_	102.22	102.37	*—*	—	2
QXPAK	3	ADG_F_	107.99	107.99	*—*	—	3
QXPAK, BI	3	ADG_F_	109.07	110.88	*NDUFAF6*,* TP53INP1*	Mitochondrial respiration, oxidative stress response	4
QXPAK	3	ADG_F_	113.46	113.46	*—*	—	5
QXPAK	5	ADG_F_	9.07	9.07	*FTO*,* AKTIP*	Growth	6
QXPAK	5	ADG_F_	18.95	18.97	*—*	—	7
QXPAK, BI	21	ADG_F_,${\bar{\text{ADRFI}}}_{F}$	7.16	9.21	*ATXN2*,* ACAD10*,* TRAFD1*,* PTPN11*	Energy homeostasis, immunity	8,17
BI	9	ADG_R_	29.66	31	*FEZF2*,* PTPRG*	Behaviour	9
BI	12	ADG_R_	99.88	99.88	*—*	—	10
QXPAK	13	ADG_R_	0.4	2.09	*RC3H1*,* TNFSF18*	Immunity	11
BI	17	ADG_R_	73.57	74.16	*LGALS3*,* TMEM260*	Circadian rhythm, immunity, lipid metabolism	12
BI	5	${\bar{\text{ADFI}}}_{F}$	3.7	3.85	*CEBPA*,* KCTD15*	Energy homeostasis, adipogenesis, feed Behaviour/food intake	13
BI	6	${\bar{\text{FCR}}}_{F}$	26.28	26.44	*SIK1B*	Hormone signaling	14
BI	16	${\bar{\text{FCR}}}_{F}$	82.86	83.26	*PLA2G4A*	Lipid metabolism, inflammatory response	15
BI	21	${\bar{\text{ADRFI}}}_{F}$	3.89	4.33	*SELENOM*	Energy metabolism	16

^1^ *Oryctolagus cuniculus* chromosome.

^2^ Annotated region, match to Table S2.

ADG_R_ showed significant associations with SNPs on OCCs 9, 12, 13 and 17 (Table 3 and Fig. 3). For this trait, however, the two models declared different chromosomal regions as significantly associated with the trait. Model 1 declared a QTL region at the proximal region of OCC 13 (0.40–2.09 Mb) containing 50 significant SNPs. The estimated SNP effect having the strongest association (minimum q‐value within the region) was 3.41 g/day. Two candidate genes (*RC3H1* and *TNFSF18*), out of 37 annotated ensembl_gene_ids, were found in this region (Table 4 and Table S2). For the same trait, model 2 declared significant signals on OCCs 9 (29.66–31.00 Mb), 12 (99.88 Mb) and 17 (73.57 Mb–74.16 Mb). The SNP effects of the QTL regions on OCCs 9 and 17 were lower (approximately between 1 and 1.5 g/day) than that detected on OCC 12, for which an effect of 3.73 g/day was estimated, which was a similar magnitude to the effects of the SNPs associated with ADG_F_. In these regions, 74 ensembl_gene_ids were annotated (Table S2) and four candidate genes were proposed (*FEZF2* and *PTPRG* on OCC 9, and *LGALS3* and *TMEM260* on OCC 17; Table 4).

The GWAS conducted with model 2 for the cage performance traits, ${\bar{\text{ADFI}}}_{F}$, ${\bar{\text{FCR}}}_{F}$ and ${\bar{\text{ADRFI}}}_{F}$, declared six significantly associated regions on OCCs 5, 6, 16 and 21 (Table 3 and Fig. 4). Region 3.70–3.85 Mb on OCC 5 was declared to be associated with ${\bar{\text{ADFI}}}_{F}$ and comprised 12 significant SNPs; that with the strongest association had a MAF of 0.37 and an estimated effect equal to 0.85 g/day. In this region, 20 ensembl_gene_ids were annotated (Table S2) and two candidate genes for ${\bar{\text{ADFI}}}_{F}$ were identified (*CEBPA* and *KCTD15*) (Table 4). For ${\bar{\text{FCR}}}_{F}$, two significant signals were detected on OCCs 6 (26.28–26.44 Mb) and 16 (82.86–83.26 Mb). The estimated effects of the SNPs with the strongest statistical association within these regions were large – 0.47 and 0.52 feed conversion units ((grams of feed/day)/(grams of growth/day)) respectively. The most significant SNP on OCC 6 had a low MAF (0.06), whereas that of the most significant SNPs on the region of OCC 16 was much higher (0.42). For ${\bar{\text{FCR}}}_{F}$, 63 ensembl_gene_ids were annotated (Table S2) and two candidate genes – salt inducible kinase 1B (putative) (*SIK1B*) on OCC 6 and phospholipase A2 group IVA (*PLA2G4A*) on OCC 16 (Table 4) – were retained.

Finally, for the last studied FE trait, ${\bar{\text{ADRFI}}}_{F}$, significant associations were detected in three regions of OCC 21: 3.89–4.33 and 7.16–7.70 Mb, and one single SNP at 9.21 Mb. The second region was particularly relevant as it also contained SNPs significantly associated with ADG_F_. The MAF of the most significant SNP within this region was 0.37, and its estimated effect was 2.16 g/day. A total of 146 ensembl_gene_ids were annotated on OCC 21 (Table S2), and the same candidate genes as those previously proposed for ADG_F_ in this region were retrieved for ${\bar{\text{ADRFI}}}_{F}$ (Table 4).

## Discussion

To our knowledge, this is the first GWAS for growth and feed efficiency traits performed in a rabbit population using a dense SNP chip panel. Our study, in addition, also introduces a new modeling approach allowing study of the association of traits recorded as group averages, when not all of the individuals in the group have been genotyped. This methodology was originally proposed for gene‐assisted selection when a certain percentage of the candidates have not been genotyped for the major gene of interest (Legarra & Vitezica, 2015). Modelling the SNP allele content using animal models has also been proposed as a tool to detect low‐quality SNPs within the panels (Forneris *et al*. 2015), an SNP being declared as erroneously genotyped when its heritability estimate is significantly different from 1. With this work, we extend the scope of application of such models to GWASs, in particular, to GWASs on group average performance traits. Previous studies (Zhang *et al*. 2018) have addressed the problem in the context of experiments where the limiting factor is the capability to generate individual phenotypes, but all of the individuals in the design were genotyped. In this case, it has been shown that pooling individual records to produce pool phenotypes and then explaining these pooled data by the mean genotype of the group produced considerable gains in the power of statistical tests over simple random sampling, i.e. random selection of as many individual phenotypes as pools were defined. This result could be expected as in the analyses of the pooled phenotypes all of the available genotypes are included, whereas in the study of a random sample of individual records only a subset of them are considered, and this sampling is particularly sensitive to low‐frequency markers. Our study, although related to the aforementioned problem, has a completely different motivation: on the one hand, there is no individual alternative to the group average phenotype recorded, and on the other hand, the experimental limitations constrain the number of genotyped animals to only a few of those responsible for the group average phenotype. In this situation, a much smaller amount of information is available for the analysis, and thus, lower power would be expected. We do not formally assess the efficiency of our proposed model, but its limited power seems obvious. On this regard, Sánchez et al. (2018) reported an important reduction in the capability to detect simulated QTL regions for one trait (out of three) that is considered as a group average with respect to the situation in which all traits are studied as individual phenotypes. They implemented a multitrait Bayesian procedure similar to the single‐step association methods (Wang *et al*. 2012), which relies on derivation of SNP effects from genomic predictions using a multiple regression in which the SNP effects are treated as random factors.

A possible means of validation of the results from the proposed bivariate model is to analyze those traits individually recorded with the two approaches. For the case of ADG_F_ the results obtained are partially the same, for example regions in OCC 3 are detected with both methods. However, other regions detected for this trait and all those declared for ADG_R_, which is a trait with lower heritability (Piles & Sanchez 2019), are not the same across the models. One reason for this is that the datasets used by each method are different. In the case of model 1, only records from genotyped animals are considered, but with the bivariate model, records from both genotyped and non‐genotyped animals are jointly considered, and the pedigree is used to predict genotypes of non‐genotyped animals with records.

As stated, given the available information on the cage performance records, the expected statistical power was low. Thus, in order to allow for a certain degree of signal detection, the threshold for significance declaration was deliberately reduced to a chromosome‐wide level. In this situation, we have successfully identified 17 chromosomal regions associated with the analyzed traits. To allow comparison between traits, the estimated SNP effects within the regions can be expressed relative to their estimated phenotypic variance (Table 2). To this end, we approximated the additive genetic variance associated with each QTL region, defined in Table 3, by considering the SNP effect with the strongest association (minimum *q*‐value) within the region and its frequency. The additive variances of the QTL regions in Table 3 represent 5–8, 0.5–8 and 0.5–2% of the phenotypic variance of ADG_F_, ADG_R_, and both ${\bar{\text{ADFI}}}_{F}$ and ${\bar{\text{ADRFI}}}_{F}$ respectively. The percentage of phenotypic variance explained by the additive genetic effect for one of the QTL regions declared for ${\bar{\text{FCR}}}_{F}$ is particularly high – 65% for the region on OCC 16. It could be difficult to propose a validation method for these results free from the assumptions in the model for the analysis, because ${\bar{\text{FCR}}}_{F}$ is recorded as the group average. Nevertheless, a simple regression of group ${\bar{\text{FCR}}}_{F}$ on group average genotype for the SNP AX‐147107945 at bp 82858725 in OCC 16, the SNP with the strongest association within the region (Table 3), also showed a strong magnitude – 0.20 (0.05) FCR units per unit of change on the cage average genotype (*P* = 3.38 × 10^−5^). This means that the expected FCR in a cage with all of the animals heterozygous for this SNP will be 0.20 units larger than that in a cage with all of the animals homozygous of one type and 0.20 units lower than that in a cage with all of the animals homozygous of the other type.

Twenty candidate genes, located in 12 QTL regions, have been proposed to explain the phenotypic variation of the traits under study; this proposition was done based on their biological functions. It is worth mentioning the *FTO* gene, annotated on OCC 5 for ADG_F_, which has been previously associated with growth traits in rabbits (Zhang *et al*. 2013; Zhang *et al*. 2014). Furthermore, *NDUFAF6*, which was also annotated for ADG_F_ in a region of OCC 3, has recently been described as a candidate gene for growth‐related traits in pigs (Ji *et al*. 2019).

It is also relevant to highlight *ATXN2*, acyl‐CoA dehydrogenase family member 10 (*ACAD10*), TRAF‐type zinc finger domain containing 1 (*TRAFD1*) and protein tyrosine phosphatase non‐receptor type 11 (*PTPN11*) genes as they map in a region of OCC 21 with a pleiotropic effect for both ADG_F_ and ${\bar{\text{ADRFI}}}_{F}$ and have 11 significant SNPs located in their introns. Identifying pleiotropic regions for both ADG_F_ and ${\bar{\text{ADRFI}}}_{F}$ could be considered an unexpected result as ${\bar{\text{ADRFI}}}_{F}$ is a trait obtained after the phenotypic correction of FI by growth and metabolic weight. However, this phenotypic correction does not grant a null genetic correlation, and in fact, in the population under study it has been reported that the genetic correlation between ADG_F_ and ${\bar{\text{ADRFI}}}_{F}$ was 0.58 (Piles & Sanchez 2019).

Piles & Sanchez (2019) showed that growth recorded in animals fed under restriction (ADG_R_) is a trait genetically different from growth recorded in animals fed *ad libitum*. Our results could be said to support this as for ADG_R_ we have declared chromosomal regions different from those declared for ADG_F_. Nonetheless, this could be also a simple consequence of our reduced statistical power. In these regions, candidate genes associated with behavioral patterns (*FEZF2*, *PTPRG* and *LGALS3*) or involved in immunity and/or lipid metabolism (*RC3H1*, *TNFSF18* and *TMEM260*) were identified. Finally, it is worth highlighting the CCAAT enhancer‐binding protein alpha (*CEBPA*) gene, annotated on OCC 5 for ${\bar{\text{ADFI}}}_{F}$, which has recently been identified as an upstream regulator of several differentially expressed genes down‐regulated in adipose tissue of high‐feed‐efficiency pigs (Horodyska *et al*. 2019).

In spite of our loose significance threshold setting, we feel relatively confident of having adequately controlled the rate of false‐positive signals that we have declared. In support of our results, we have identified some candidate genes that have already been associated with similar traits in other rabbit and pigs populations.

## Conclusions

We have proposed a number of QTL regions linked to the observed variation of the studied traits using a complex statistical model for fitting cage FE and feed intake, jointly with individual genotypes. To our knowledge, this is the first time this type of statistical model has been used within the framework of GWAS studies. The information content on cage average performances is quite limited, thus we have reduced the threshold for significance declaration to a chromosome‐wide level. In spite of this loose significance threshold definition, the declared QTL seem to harbor genes that can clearly be regarded as functional candidates for the traits of interest. Our results seem to support the idea that the growth of animals fed on restriction is under a different genetic control that that of animals fed *ad libitum* as we have identified different QTL regions for both traits. It is remarkable that genes related to behavioral patterns have been proposed as candidates for ADG_R_. Regarding FE, some of the QTL regions that we declared to harbor candidate genes which are involved in lipid and energy metabolism have a pleiotropic effect for both ADG_F_ and ${\bar{\text{ADRFI}}}_{F}.$ In spite of these promising results, further functional research is warranted to validate these genes. Overall, our results lay an important foundation for future studies to unravel the underlying genetic bases driving growth and FE regulation in rabbits.

## Availability of data

Genotypes can be found here: https://doi.org/10.5281/zenodo.3611097

## Supporting information


**Table S1.** Description of the 189 chromosome‐wise significant SNPs and features annotated to them.

**Table S2.** List of genes annotated in the QTL regions.

## References

[age12988-bib-0001] Bindea G. , Mlecnik B. , Hackl H. *et al* (2009) ClueGO: A Cytoscape plug‐in to decipher functionally grouped gene ontology and pathway annotation networks. Bioinformatics 25, 1091–3. 19237447 10.1093/bioinformatics/btp101 PMC2666812

[age12988-bib-0002] Carneiro M. , Rubin C.J. , Di Palma F. *et al* (2014) Rabbit genome analysis reveals a polygenic basis for phenotypic change during domestication. Science 345, 1074–9. 25170157 10.1126/science.1253714 PMC5421586

[age12988-bib-0003] Cartuche L. , Pascual M. , Gómez E.A. *et al* (2014) Economic weights in rabbit meat production. World Rabbit Science 22, 165–77.

[age12988-bib-0004] Cesari V. , Zucali M. , Bava L. , Gislon G. , Tamburini A. , Toschi I. (2018) Environmental impact of rabbit meat: The effect of production efficiency. Meat Science 145, 447–54. 30055437 10.1016/j.meatsci.2018.07.011

[age12988-bib-0005] Chang C.C. , Chow C.C. , Tellier L.C. , Vattikuti S. , Purcell S.M. , Lee J.J. (2015) Second‐generation PLINK: rising to the challenge of larger and richer datasets. Gigascience 4(1), 7 10.1186/s13742-015-0047-8 25722852 PMC4342193

[age12988-bib-0006] Drouilhet L. , Achard C.S. , Zemb O. *et al* (2016) Direct and correlated responses to selection in two lines of rabbits selected for feed efficiency under ad libitum and restricted feeding: I. Production traits and gut microbiota characteristics. Journal of animal science 94, 38–48. 26812310 10.2527/jas.2015-9402

[age12988-bib-0007] Emmerson D.A. (1997) Commercial approaches to genetic selection for growth and feed conversion in domestic poultry. Poultry Science 76, 1121–5. 10.1093/ps/76.8.1121 9251138

[age12988-bib-0008] Eppig J.T. , Smith C.L. , Blake J.A. , Ringwald M. , Kadin J.A. , Richardson J.E. , Bult C.J. (2017) Mouse Genome Informatics (MGI): resources for mining mouse genetic, genomic, and biological data in support of primary and translational research. Systems Genetics 2017, 47–73. 10.1007/978-1-4939-6427-7_3 27933520

[age12988-bib-0009] Estany J. , Camacho J. , Baselga M. , Blasco A. (1992) Selection response of growth rate in rabbits for meat production. Genetics Selection Evolution 24, 527.

[age12988-bib-0010] Forneris N.S. , Legarra A. , Vitezica Z. , Tsuruta S. , Aguilar I. , Misztal I. , Cantet R.J.C. (2015) Quality control of genotypes using heritability estimates of gene content at the marker. Genetics 199, 675–81. 25567991 10.1534/genetics.114.173559 PMC4349063

[age12988-bib-0011] Garreau H. , Ruesche J. , Gilbert H. *et al* (2019) Estimating direct genetic and maternal effects affecting rabbit growth and feed efficiency with a factorial design. Journal of Animal Breeding and Genetics 136, 168–73. 30687950 10.1111/jbg.12380

[age12988-bib-0012] Gidenne T. , Combes S. , Fortun‐Lamothe L. (2012) Feed intake limitation strategies for the growing rabbit: effect on feeding behaviour, welfare, performance, digestive physiology and health: a review. Animal 6, 1407–19. 23031513 10.1017/S1751731112000389

[age12988-bib-0013] Gidenne T. , Fortun‐Lamothe L. , Bannelier C. *et al* (2017) Direct and correlated responses to selection in two lines of rabbits selected for feed efficiency under ad libitum and restricted feeding: III. Digestion and excretion of nitrogen and minerals. Journal of Animal Science 95, 1301–12. 28380512 10.2527/jas.2016.1192

[age12988-bib-0014] Gómez E.A. , Rafel O. , Ramon J. (2002) The Caldes strain Rabbit genetic resources in mediterranean countries. Options Méditerranéennes: Série B. Etudes et Recherches 38, 189–98.

[age12988-bib-0015] Horodyska J. , Reyer H. , Wimmers K. , Trakooljul N. , Lawlor P.G. , Hamill R.M. (2019) Transcriptome analysis of adipose tissue from pigs divergent in feed efficiency reveals alteration in gene networks related to adipose growth, lipid metabolism, extracellular matrix, and immune response. Molecular Genetics and Genomics 294, 395–408. 30483895 10.1007/s00438-018-1515-5

[age12988-bib-0016] Ji J. , Yan G. , Chen D. , Xiao S. , Gao J. , Zhang Z. (2019) An association study using imputed whole‐genome sequence data identifies novel significant loci for growth‐related traits in a Duroc × Erhualian F_2_ population. Journal of Animal Breeding and Genetics 136, 217–28. 30869175 10.1111/jbg.12389

[age12988-bib-0017] Kang H.M. , Sul J.H. , Zaitlen N.A. *et al* (2010) Variance component model to account for sample structure in genome‐wide association studies. Nature Genetics 42, 348–54. 20208533 10.1038/ng.548 PMC3092069

[age12988-bib-0018] Legarra A. , Vitezica Z. (2015) Genetic evaluation with major genes and polygenic inheritance when some animals are not genotyped using gene content multiple‐trait BLUP. Genetics Selection Evolution 47, 89. 10.1186/s12711-015-0165-x PMC5518164 26576649

[age12988-bib-0019] Lindblad‐Toh K. , Garber M. , Zuk O. *et al* (2011) A high‐resolution map of human evolutionary constraint using 29 mammals. Nature 478, 476. 21993624 10.1038/nature10530 PMC3207357

[age12988-bib-0020] McLaren W. , Gil L. , Hunt S.E. *et al* (2016) The ensembl variant effect predictor. Genome Biology 17, 122. 27268795 10.1186/s13059-016-0974-4 PMC4893825

[age12988-bib-0021] Pérez‐Enciso M. , Misztal I. (2011) Qxpak5: Old mixed model solutions for new genomics problems. BMC Bioinformatics 12, 202. 21612630 10.1186/1471-2105-12-202 PMC3123239

[age12988-bib-0022] Piles M. , Sánchez J.P. (2019) Use of group records of feed intake to select for feed efficiency in rabbit. Journal of Animal Breeding and Genetics 136, 474–83. 31020712 10.1111/jbg.12395

[age12988-bib-0023] Piles M. , David I. , Ramon J. , Canario L. , Rafel O. , Pascual M. , Ragab M. , Sánchez J.P. (2017) Interaction of direct and social genetic effects with feeding regime in growing rabbits. Genetics Selection Evolution 49, 58. 10.1186/s12711-017-0333-2 PMC5520409 28728597

[age12988-bib-0024] Safran M. , Solomon I. , Shmueli O. *et al* (2002) GeneCards™ 2002: towards a complete, object‐oriented, human gene compendium. Bioinformatics 18, 1542–3. 12424129 10.1093/bioinformatics/18.11.1542

[age12988-bib-0025] Sánchez J.P. , Legarra A. , Piles M. (2018) Multiple trait single step Bayesian GWAS on pooled data. Proceedings of the World Congress on Genetics Applied to Livestock Production, 11.275 (http://www.wcgalp.org/proceedings/2018/multiple‐trait‐single‐step‐bayesian‐gwas‐pooled‐data)

[age12988-bib-0026] Sather A.P. , Fredeen H.T. (1978) Effect of selection for lean growth rate upon feed utilization by the market hog. Canadian Journal of Animal Science 58, 285–9.

[age12988-bib-0027] Smedley D. , Haider S. , Durinck S. *et al* (2015) The BioMart community portal: an innovative alternative to large, centralized data repositories. Nucleic Acids Research 43, 589–98. 10.1093/nar/gkv350 PMC4489294 25897122

[age12988-bib-0028] Storey J.D. (2002) A direct aproach to false discovery rates. Journal of the Royal Statistical Society: Series B (Statistical Methodology) 64, 479–98.

[age12988-bib-0029] Storey JD , Bass AJ , Dabney A , Robinson D (2019) qvalue: Q‐value estimation for false discovery rate control. R package version 2.18.0, http://github.com/jdstorey/qvalue

[age12988-bib-0030] Wang H , Misztal I , Aguilar I , Legarra A , Muir W (2012) Genome‐wide association mapping including phenotypes from relatives without genotypes. Genetics Research 94, 73–83. 22624567 10.1017/S0016672312000274

[age12988-bib-0031] Zhang GW , Gao L , Chen SY *et al* (2013) Single nucleotide polymorphisms in the FTO gene and their association with growth and meat quality traits in rabbits. Gene 527, 553–7. 23796799 10.1016/j.gene.2013.06.024

[age12988-bib-0032] Zhang G.W. , Jia W. , Chen S.Y. , Jia X.B. , Wang J. , Lai S.J. (2014) Association between the IRS1 and FTO genes regulates body weight in rabbits. Gene 548, 75–80. 25014140 10.1016/j.gene.2014.07.011

[age12988-bib-0033] Zhang W. , Liu A. , Albert P.S. , Ashmead R.D. , Schisterman E.F. , Mills J.L. (2018) A pooling strategy to effectively use genotype data in quantitative traits genome‐wide association studies. Statistics in Medicine 37, 4083–95. 30003569 10.1002/sim.7898 PMC6204292

